# Convective Heat Transfer in Magneto-Hydrodynamic Carreau Fluid with Temperature Dependent Viscosity and Thermal Conductivity

**DOI:** 10.3390/nano12224084

**Published:** 2022-11-20

**Authors:** Syed Amir Ghazi Ali Shah, Ali Hassan, Najah Alsubaie, Abdullah Alhushaybari, Fahad M. Alharbi, Ahmed M. Galal, Diana-Petronela Burduhos-Nergis, Costica Bejinariu

**Affiliations:** 1 Department of Mathematics, Capital University of Science and Technology, Islamabad 46000, Pakistan; 2 Department of Mathematics, University of Gujrat, Gujrat 50700, Pakistan; 3 Department of Computer Sciences, College of Computer and Information Sciences, Princess Nourah bint Abdulrahman University, P.O. Box 84428, Riyadh 11671, Saudi Arabia; 4 Department of Mathematics, College of Science, Taif University, P.O. Box 11099, Taif 21944, Saudi Arabia; 5 Department of Mathematics, Al-Qunfudah University College, Umm Al-Qura University, Mecca 28821, Saudi Arabia; 6 Department of Mechanical Engineering, College of Engineering in Wadi Alddawasir, Prince Sattam bin Abdulaziz University, Al-Kharj 16278, Saudi Arabia; 7 Production Engineering and Mechanical Design Department, Faculty of Engineering, Mansoura University, Mansoura P.O. 35516, Egypt; 8 Faculty of Materials Science and Engineering, “Gheorghe Asachi” Technical University, 700050 Iasi, Romania

**Keywords:** carreau fluid, convective boundary, stretching/shrinking, porous medium and variable viscosity and thermal conductivity

## Abstract

This study is aimed to explore the magneto-hydrodynamic Carreau fluid flow over a stretching/shrinking surface with a convectively heated boundary. Temperature-dependent variable thermophysical properties are utilized to formulate the problem. The flow governing equations are obtained with boundary layer approximation and constitutive relation of the Carreau fluid. The shooting method is utilized to obtain graphical and numeric outcomes. Additionally, initial guesses are generated with the help of Newton’s method. The effect of Weissenberg number, Magnetization, stretching ratio, Prandtl number, suction/blowing parameter, and Lewis number is obtained on velocity, temperature and species continuity profile and analyzed. Shear stress rates and Nusselt number outcomes under body forces influences are present in tabulated data and discussed. It is observed that in absence of magnetization force, *B* = 0 and strong mass suction 5≤S≤7.5 effect high rates of Nusselt number is obtained. It is concluded that under the influence of power law index and non-linearity parameter maximum heat transfer and reduced shear stress rates are obtained.

## 1. Introduction

Heat transfer enhancement using different physical configurations and fluids has been an interesting topic for researchers over the last two decades. Many scientists have given much attention to this field due to its wide range of applications in industrial and engineering processes. Production of rubber, colloidal suspension of fluid, glass production, and spinning of metal are some examples of its applications. Likewise, the term “nanofluid” was first coined by Chio and Eastman in the year 1995. They discovered in their research how to increase the thermal conductivity of base fluid by dispersing metallic or non-metallic non-meter-sized particles in it. The formed mixture was given the name nanofluid. The thermal conductivity of the formed mixture was found to be enhanced. Recently, another category of fluids has surfaced known as “hybrid nanofluids”. In these types of fluids, instead of one, two nano-meter sized nano-particles, metallic or non-metallic, are dispersed into the host base liquid. This innovation has immensely enhanced the thermal conductivity of fluids consequently as a result high heat transfer rates and reduced shear stress rates are achieved. Mandal et al. [1] discussed convective heat transfer in micro-polar nanofluid over non-linear vertical stretching. Pal and Mandal [2] investigated thermal radiative heat transfer with variable properties over plate in porous medium.

Hiemenz [3] studied orthogonal flow stagnation flow over a two-dimensional stationary flat plate. Erickson et al. [4] explained the boundary layer incompressible fluid flow over an in-extensible at surface with uniform velocity. Olajuwan [5] examined Carreau fluid with transfer of mass and heat with magneto-hydrodynamics effect through a porous medium. Hayat et al. [6] solved various wave forms Carreau liquid in MHD peristaltic flow in a channel. Akbar et al. [7] studied the numerical simulation of 2-D tangent hyperbolic flow of fluid through an extending sheet placed in a magnetic field. Suneetha and Gangadhar [8] examined MHD effect and convectively heated boundary over stretching sheet. Abou Zeid et al. [9] conducted statistical analysis of Carreau MHD flow with heat and mass transfer in the presence of non-Darcy decomposition and chemical reaction. Akram et al. [10]. discussed MHD peristaltic flow of Carreau nano-fluid in an asymmetric channel.

Prilepskii et al. [11] employed Urokinase conjugate magnetite nano-particles as an effective drug delivery tool. They investigated these nano-particles to investigate thrombolysis synthesis and pre-clinical evaluation. Kurlyandskaya et al. [12] discussed fabrication, characterization and bio-compatibility with electrostatic of water-based suspensions of Iron oxide nano-particles. Spizzo et al. [13] provided the synthesis of ferro-fluids formed of iron oxide nano-flowers. They elaborated on the interplay between the carrier fluid and magnetic particles. Bender et al. [14] introduced synthesis and characterization of ferro-gels with Ni as ferro phase. The detection of blood clot and position of blood clot are the major application of ferro-gels. Buznikov et al. [15] described modelling of magneto impedance response of thin film sensitive in the presence of ferro-gels. They provided significant insight into development of biosensor of for tissue embedded in magnetic nano-particle detection. Jain and Grimes [16] introduced wireless magneto-elastic micro sensor to measure temperature and pressure simultaneously. Pal et al. [17] examined mixed convection over stretching/shrinking sheet with viscous dissipation and heat source/sink using nanofluids.

Akbar and Nadeem [18] investigated Carreau fluid and analyzed heat and mass transfer in peristaltic flow taking into account the long wavelengths. Nandeppanavar et al. [19] studied heat transfer in MHD fluids over stretching the surface under the impact of variable thermal conductivity and non-uniform heat source. Cortell [20] discussed in-compressible viscid flow on a nonlinear stretchable surface with heat transfer. Vyas and Ranjan [21] explored thermal radiation and viscous dissipation effects on MHD flow and heat transfer over nonlinear stretching surface. Ali et al. [22] demonstrated the effect of suction/injection on the stretching surface with power law flow. Sandeep et al. [23] examined semiconductor vertical porous panels under radiative and chemical reaction effect to analyze heat transfer in an unstable flow. Shen et al. [24] described MHD stagnation point flow with respect to the leaky leaf on the grid at a slip speed. Akyildiz and Siginer. [25] provided analytical solutions using the spectral Galerkin Legend process for viscous fluid over a nonlinear stretchable surface. Bhattacharyya et al. [26] studied stagnation point flow of in-compressible fluids under the effect of partial slips over shrinking surface. Chen et al. [27] investigated non-Newtonian flow with viscous dissipation over nonlinear stretching surface. In the recent years numerous researchers investigated the different flow regimes over stretching sheet, here some of the notable studies are mentioned [28,29,30].

Abdou and El-Zahar [31] demonstrated the effect of variable thermo-physical properties on micro polar fluid with heat generation. Salem and Odda [32] studied suction or injection effect on the micro-polar fluid with variable thermal conductivity and viscosity. Uwanta and Usman [33] discussed the impact of variable thermo-physical properties on a volatile flow of the board layer in the presence of a magnetic chip in the micro-polar liquid. Abd El-Hakiem et al. [34] elaborated natural convection MHD micro polar fluid with the effect of variable viscosity. Pal and Mandal [35,36] discussed Sisko stagnation point flow with suction and analyzed effect of aligned magnetic field on heat transfer with heterogeneous-homogeneous reaction in carbon nanotube water-based flow. Linearly stretching surfaces with different flow regimes has been extensively examined to analyze distinct body force effect, some of the notable works are given for the knowledge gains [37,38,39,40,41].

Devi and Kandasmy [42] analyzed laminar flow under impact of homogeneous chemical reaction over semi-infinite horizontal plate with heat transfer. Chamkha and Rashad [43] discussed chemical reactions in the presence of heat generation or absorption in MHD flow over uniform permeable surface. Mabood at al. [44] presented radiation, viscous dissipation and chemical reaction effects on MHD heat and mass transfer of nanofluids within the porous medium. Raptis and Perdikis [45] observed the viscid flow on a nonlinear stretchable sheet in the presence of magnetic field parameter by applying shooting technique. The impact of slip boundary condition on heat transfer rate were investigated by Das et al. [46]. Through their study they discovered that in the presence of thermal slip condition or hydrodynamic suction, injection parameter has large impact on surface temperature of plate. At high absolute temperature levels, the thermal radiation effect becomes intensified. Hussain et al. [47,48,49,50] provided significant insight into the hybrid nanofluids over rotating flow mechanism.

Salahuddin et al. [51] investigated activation energy and heat generation effect on Carreau fluid with variable properties using RK-4 and shooting method. Rehman et al. [52] presented theoretical analysis of Carreau for multiple flow regimes using shooting method. Salahuddin and Awais [53] discussed Cross and Carreau fluid with variable thermophysical properties employing shooting method. Hussain et al. [54] studied non-uniform heat generation in Carreau fluid across nonlinear elongating cylinder utilizing shooting method. Yang et al. [55] examined multiple solution stagnation point flow of Carreau fluid with non-uniform heat generation using RK-4 and shooting method. Mandal and Pal [56,57] analyze entropy generation with magneto-hydrodynamic effect in hybrid nanofluids, carbon nanotubes flow nonlinear radiation effect and variable thermophysical properties.

The current study is aimed to examine convective heat transfer in classical non-Newtonian Carreau fluid under influence of variable thermophysical properties.

Rehamn et al. [52] conducted group theoretic analysis of Carreau fluid they employed constant thermophysical properties. Recently, Salahuddin and Awais [53] presented comparative study of non-Newtonian fluids by only incorporating variable thermal conductivity. Hussain et al. [54] examined Carreau fluid over nonlinear elongating cylinder for thermal characterization. Yang et al. [55] introduced stagnation point multiple solutions of unsteady Carreau fluid. The novelty of this work is to investigate convective heat transfer over permeable nonlinear stretching/shrinking sheet. The purpose is to implement the variable thermo-physical properties impact and magneto-hydrodynamic effect on flow of Carreau flow in the presence of convective condition. The system of flow equations is obtained with the help of boundary layer theory and constitutive relation of Carreau fluid. Additionally, the achieved flow guiding equations are converted into first order ordinary differential equations with our own supposed notations. Additionally, this problem is solved with shooting technique in MATLAB and the Newton’s method is employed to obtain the initial guesses for the problem. Furthermore, analysis of achieved graphical and numeric results is conducted for different study profiles namely: velocity, temperature and concentration. The influence of different parameters is depicted on drag coefficient and Nusselt number presented in tabulated data sets.

## 2. Mathematical Formulation

### 2.1. Statement of Problem

Consider a non-linearly stretchy sheet with a uniform 2-D in-compressible viscid flow of an electrically conducting fluid. Meanwhile, the plate has been stretched with the velocity $u_{w}=ax^{m}$ along *x*-direction. Here $T_{w}$ is the wall temperature and $C_{w}$ is the nano-particles concentration at the stretching sheet, $T_{\infty }$ is the free stream temperature and $C_{\infty }$ is the ambient concentration. The two-dimensional governing equations, which include the continuity, momentum, energy, and concentration equations, are used to explain the flow.

Equation of continuity [52,55]

(1) $$\frac{\partial u}{\partial x}+\frac{\partial v}{\partial y}=0.$$

Momentum Equations

(2) $$u\frac{\partial u}{\partial x}+v\frac{\partial u}{\partial y}=\frac{1}{\rho }\frac{\partial }{\partial y}\left[\mu \left(T\right)\frac{\partial u}{\partial y}\right]+3v_{f}\frac{n-1}{2}\Gamma {\left(T\right)}^{2}{\left(\frac{\partial u}{\partial y}\right)}^{2}\frac{\partial^{2}u}{\partial y^{2}}+\frac{\sigma }{\rho }J^{2}\left(u_{e}-u\right)+u_{e}\frac{\partial u_{e}}{\partial x}.$$

Energy Equation without *q_r_* relation [52,55]

(3) $$u\frac{\partial T}{\partial x}+v\frac{\partial T}{\partial y}=\frac{k_{f}\left(T\right)}{{\left(\rho c_{p}\right)}_{f}}\left[\frac{\partial^{2}T}{\partial y^{2}}\right].$$

Species continuity equation

(4) $$\frac{v\partial C}{\partial y}+\frac{u\partial C}{\partial x}=D\frac{\partial^{2}C}{\partial y^{2}}.$$

The dynamic viscosity is represented by μ, and the thermal conductivity is represented by $k_{f}$. Furthermore, the electrical conductivity, kinematic viscosity, and fluid density are denoted by $\sigma ,\;v_{f}\;and\;\rho$, respectively. The symbol $c_{p}$ denotes specific heat capacity. *T* stands for fluid temperature, $T_{\infty }$ for free stream temperature, and *D* denotes thermal diffusivity. Temperature dependent thermal conductivity and viscosity have major possibilities in such a field as magneto-electro resonance [16]. We are using variable viscosity and thermal conductivity, therefore, the variable relations for both said thermo-physical properties are given as follows:

(5) $$\mu \left(T\right)=\mu^{*}\left[N_{1}-h_{1}\left(T_{\infty }-T\right)\right],\;k_{f}\left(T\right)=k_{f}{}^{*}\left[N_{2}-h_{2}\left(T_{\infty }-T\right)\right].$$

The effect of variable thermal conductivity and viscosity are represented by $\mu^{*}$ and $k_{f}^{*}$ in the preceding equation, whereas $h_{1},\;h_{2},\;N_{1}\;and\;N_{2}$ are some positive constants. In addition, the values $N_{1}\;and\;N_{2}$ are set to 1. The associated boundary conditions are taken as [52,55]:

(6) $$u=u_{w}\left(x\right)=ax^{m},\;v=v_{w}\left(x\right),\;\;\frac{\partial T}{\partial y}=-\frac{q_{w}\left(x\right)}{k_{f}},\;C=C_{w}\;\;\;at\;y=0,\;\;\;u\to u_{e}\left(x\right)=bx^{m},\;T\to T_{\infty },C\to C_{\infty },\;\;\;at\;\;y\to \infty .$$

**Figure 1:**
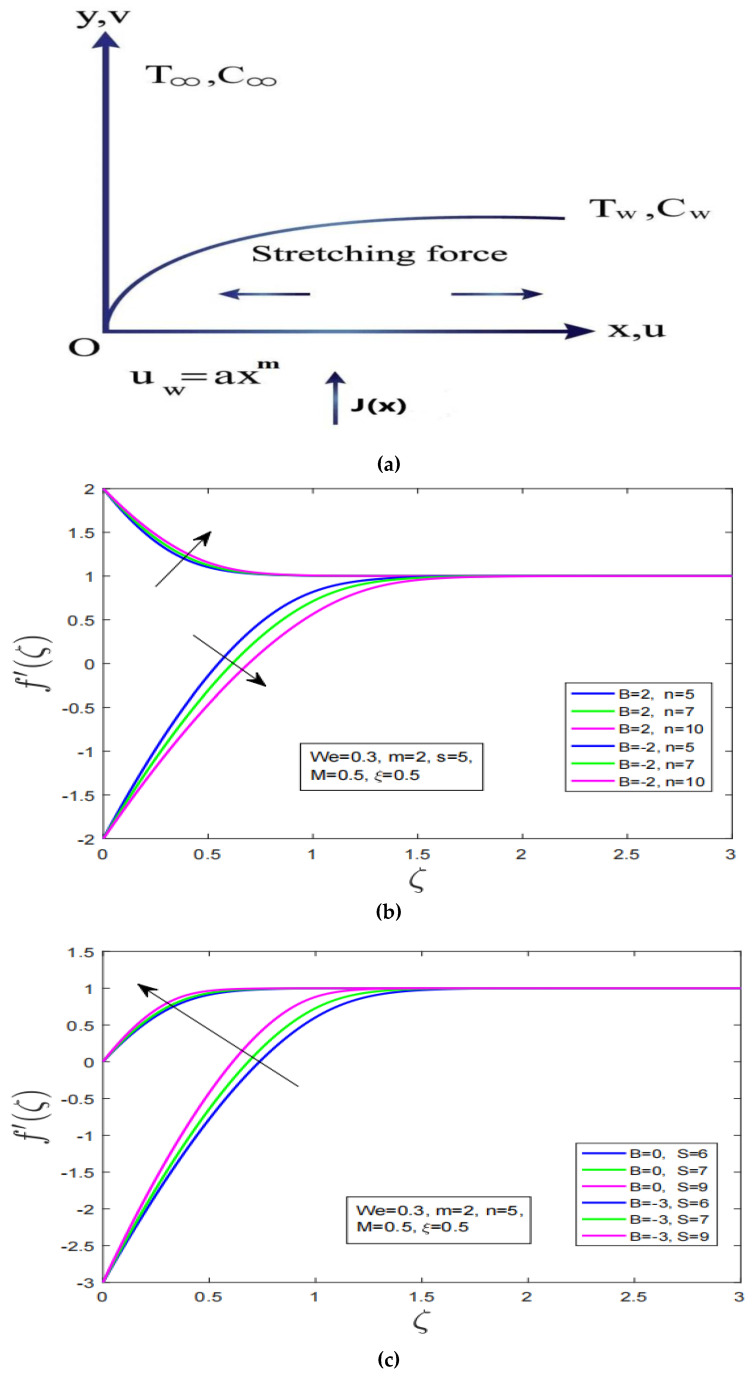

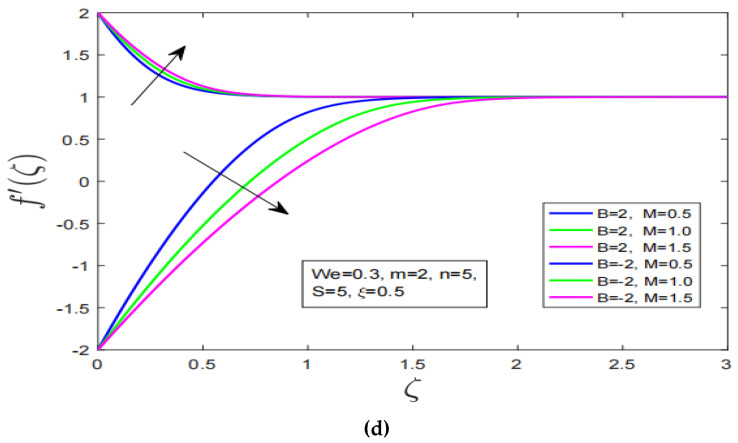
(**a**): Systematic representation of Physical model, (**b**): Effect of power law index on velocity profile over stretching and shrinking surface, (**c**): Impact of mass suction effect on velocity profile over stretching and shrinking surface, (**d**): Influence of magnetization on velocity profile over stretching and shrinking surface.

Here, $v_{w}$ stand for injection/suction velocity and $q_{w}$ is the surface heat flux. Figure 1a below shows the configuration and coordinates system of the problem.

### 2.2. Transformation of Flow Governing Equations

In this section the flow governing Equations (1)–(4) along with boundary conditions (6) are converted into dimensionless equations. For this purpose, we define the following similarity transformations:

(7) $$\{\begin{array}{c} \theta \left(\eta \right)=\frac{T-T_{w}}{\left(T_{\infty }-T_{w}\right)}\\ \phi \left(\eta \right)=\frac{C-C_{w}}{\left(C_{\infty }-C_{w}\right)} \end{array}\;,\;\varphi ={\left(bv\right)}^{1/2}x^{\frac{m+1}{2}}f\left(\zeta \right),\;\zeta ={\left(\frac{b}{v}\right)}^{1/2}yx^{\frac{m-1}{2}}.$$

where, φ is a stream function. Now, after utilizing the above defined similarity transform and stream function the transform flow governing equations are as follows:

(8) $$\begin{array}{cc} f^{\prime \prime \prime }\left(\zeta \right)+m(1- & {f^{\prime }}^{2}\;\left(\zeta \right))+\frac{m+1}{2}f\left(\zeta \right)f^{\prime \prime }\left(\zeta \right)+\xi f^{\prime \prime \prime }\left(\zeta \right)-\xi \left(f^{\prime \prime }\left(\zeta \right)\theta^{\prime }\left(\zeta \right)+f^{\prime \prime \prime }\left(\zeta \right)\theta \left(\zeta \right)\right)+\frac{3}{2}(n\\ & -1)We^{2}{f^{\prime \prime }}^{2}\;\left(\zeta \right)f^{\prime \prime \prime }\left(\zeta \right)M^{2}\left(1-f^{\prime }\left(\zeta \right)\right)=0, \end{array}$$

(9) $$\theta^{\prime \prime }\left(\zeta \right)+Pr\frac{m+1}{2}\theta^{\prime }\left(\zeta \right)f\left(\zeta \right)+\epsilon \left(\theta \left(\zeta \right)\theta^{\prime \prime }\left(\zeta \right)+{\theta^{\prime }}^{2}\left(\zeta \right)\right)=0,$$

(10) $$\phi^{\prime \prime }\left(\zeta \right)+Le\frac{m+1}{2}\phi^{\prime }\left(\zeta \right)f\left(\zeta \right)=0.$$

Now, converted boundary conditions are as follows:

(11) $$f\left(0\right)=S,\;f^{\prime }\left(0\right)=B,\;\theta^{\prime }\left(0\right)=-1,\;\phi \left(0\right)=1,\;as\;\zeta \to 0,\;\;\;f^{\prime }\left(\zeta \right)\to 1,\;g\left(\eta \right)\to 0,\;\theta \left(\zeta \right)\to 0,\;\phi \left(\zeta \right)\to 0\;\;as\;\;\zeta \to \infty .$$

In the above equations $We^{2}=\frac{\Gamma^{2}b^{3}x^{3m-1}}{\upsilon_{f}}$ denotes Weissenberg number, $M^{2}=\frac{\sigma J^{2}}{\rho b}$ is magnetic field parameter, $S=\frac{-2v_{w}}{{\left(bv\right)}^{1/2}\left(m+1\right)\;x^{\frac{m-1}{2}}}$ is suction/injection parameter, $B=\frac{a}{b}$ is stretching ratio, $Pr=\frac{\mu C_{p}}{k_{f}}$ describe Prandtl number, $\xi =h_{1}\left(T_{\infty }-T_{w}\right)$ is viscosity parameter, $\epsilon =h_{2}\left(T_{\infty }-T_{w}\right)$ is thermal conductivity parameter and $Le=\frac{\nu_{f}}{D}$ Lewis Number.

### 2.3. Drag Coefficient and Nusselt Number

The shear stress and heat flux are the physical quantities of engineering interest. Here, after utilizing the boundary layer approximation (BLA) and constitutive relation for Carreau fluid, following relations represent wall shear stress and wall heat flux, respectively.

(12) $$\tau_{w}=[\frac{\partial u}{\partial y}+\frac{\left(n-1\right)}{2}\Gamma^{2}(T)({\frac{\partial u}{\partial y})}^{3}]\;at\;y=0,and\;q_{w}=-k_{f}(T)[\frac{\partial T}{\partial y}]\;at\;y=0.$$

The skin friction and Nusselt number given by

(13) $$C_{f}=\frac{\tau_{w}}{ax\sqrt{a/\nu_{f}}},\;\;\;and\;Nu_{x}=\frac{xq_{w}}{k_{f}\left(T\right)\left(T-T_{\infty }\right)}.$$

Skin friction and Nusselt number in dimensionless form are defined as follows:

(14) $$\sqrt{Re_{x}}C_{f}=f^{\prime \prime }\left(0\right)+\frac{\left(n-1\right)}{2}We^{2}{\left(f^{\prime \prime }\left(0\right)\right)}^{2}\;\;\;and\;Re_{x}^{-1/2}Nu_{x}=-\frac{1}{\theta \left(0\right)}\;.$$

## 3. Method of Solution

Different numerical methods have been used by scientists over the years to obtain reliable and effective results of the problem. Abou-zeid [9] tackled their problem using the finite difference scheme for Carreau. Sandeep [23] studied the analytical solutions of the Carreau fluid. Shen [24] utilized the homotopy analysis method to study the carreau fluid for mixed convection. Arshad [34], Hassan [35] and Hussain [36,37] used the BVP-4c technique to study the stretching surface. In this study, we shall obtain the outcomes of our study using shooting method. The problem is formulated through the following steps:

1. Formulate the flow governing equation in the presence of body forces with help of
2. General Naiver-Stokes and boundary layer approximation. Additionally, utilizing the constitutive relations of Carreau fluid.
3. Transform the achieved flow governing equations into ordinary differential equations using suitable similarity transforms.
4. Introduce a set of new variables to convert the ordinary set of differential equations into an initial value problem.
5. Calculate the initial guesses using Newton’s method and simulate the problem employing shooting method.
6. Set the tolerance of numerical solution in MATLAB and Code the whole problem.
7. Finally, achieve graphical results for different profiles. Obtain numeric outcomes for shear stress rate and Nusselt number.

The following notions have been used to transform the above non dimensional flow equation along with the boundary equations.

(15) $$f=Y_{1},\;f^{\prime }=Y_{1}^{\prime }\;=Y_{2},\;\;f^{\prime \prime }=Y_{2}^{\prime }\;=Y_{3},\;f^{\prime \prime \prime }=Y_{3}^{\prime }\;,\;\theta =Y_{4},\;\theta^{\prime }=Y_{4}^{\prime }\;=Y_{5},\;\;\theta^{\prime \prime }=Y_{5}^{\prime }\;,\;\phi =Z_{1},\;\phi^{\prime }=Z_{1}^{\prime }\;=Z_{2},\;\;\phi^{\prime \prime }=Z_{2}^{\prime }.$$

The following set of ordinary differential equations are obtained by using the above set of notations.

(16) $$Y_{1}^{\prime }\;=Y_{2}$$

(17) $$Y_{2}^{\prime }\;=Y_{3}$$

(18) $$Y_{3}^{\prime }\;=\left[\frac{(-m\left(1-Y_{2}^{2}\right)-\frac{m+1}{2}Y_{1}Y_{3}+\xi Y_{3}Y_{5}-M^{2}\left(1-Y_{2}\right)}{1+\xi -\xi Y_{4}+\frac{3}{2}\left(n-1\right)We^{2}Y_{3}^{2}}\right]$$

(19) $$Y_{4}^{\prime }\;=Y_{5}$$

(20) $$Y_{5}^{\prime }\;=\left[\frac{-Pr\frac{m+1}{2}Y_{1}Y_{5}-\epsilon Y_{5}^{2}}{1+\epsilon Y_{4}}\right]$$

(21) $$Z_{1}^{\prime }\;=Z_{2}$$

(22) $$Z_{2}^{\prime }\;=-\frac{m+1}{2}LefZ_{2}.$$

The boundary conditions are defined as follows:

(23) $$Y_{1}\left(0\right)=S,\;Y_{2}\left(0\right)=B,\;Y_{3}\left(0\right)=r,\;Y_{4}\left(0\right)=q,\;Y_{5}\left(0\right)=1,\;Z_{1}\left(0\right)=1,\;Z_{2}\left(0\right)=Q.$$

The missing conditions r,q and Q assumed to satisfy the following relations:

(24) $$Y_{2}\left(\zeta_{\infty },\;r,q\right)=1,\;Y_{2}\left(\zeta_{\infty },\;r,q\right)=0,\;Z_{1}\left(\zeta_{\infty },\;Q\right)=0.$$

The Stopping criteria for the Newton’s method is set as:

(25) $$max\left\{\left|Y_{2}\left(\zeta_{\infty }\right)-1\right|,\;\;\;\left|Y_{4}\left(\zeta_{\infty }\right)\right|\right\}<\delta ,\;and\;\;\left|Z_{1}\left(\zeta_{\infty },\;Q\right)\right|<\delta .$$

## 4. Results and Discussion

Here, in this section, the outcomes of the influence of distinct study parameters are presented. Different study profiles namely: velocity, temperature and concentration are observed under the varying effect of thermal conductivity, viscosity, magnetization force, Weissenberg number, Lewis number, Prandtl number and mass suction influence for both stretching and shrinking surface. The analysis of outcomes is conducted here along with discussion on achieved outcomes. The shear stress rates and Nusselt number under the varying impression of study parameters are presented in tabulated data set and elaborated in discussions.

Figure 1b–d illustrates the impact of power law index, mass suction effect and magnetization force on velocity profile of Carreau fluid over stretching/shrinking surface, respectively. Additionally, it is worth mentioning here that *B* = 2,3 denotes the stretching in surface and *B* = −2,−3 describe the shrinking in surface. It is observed that under increment in power law index in case of stretching surface the motion of Carreau fluid has decreased. Whereas, in shrinking case the velocity has increased with augmentation in power law index. It is worth noting here that thickness of associated momentum boundary of Carreau fluid has expanded in case of shrinking while it has contracted in the case of stretching (See Figure 1b). Figure 1c, depicts the impact of mass suction on the motion profile of Carreau fluid for both stretching and shrinking surface. It is noted that with increment in mass suction the velocity has increased for stretching/shrinking. Furthermore, the magnetization does affect velocity profile for stretching/shrinking oddly. The generated Lorentz force in the vicinity of boundary layer of Carreau fluid has increased velocity for stretching/shrinking. Moreover, the associated momentum boundary layer under influence of magnetization force has increased for shrinking surface dramatically as compared to stretching surface.

**Figure 2:**
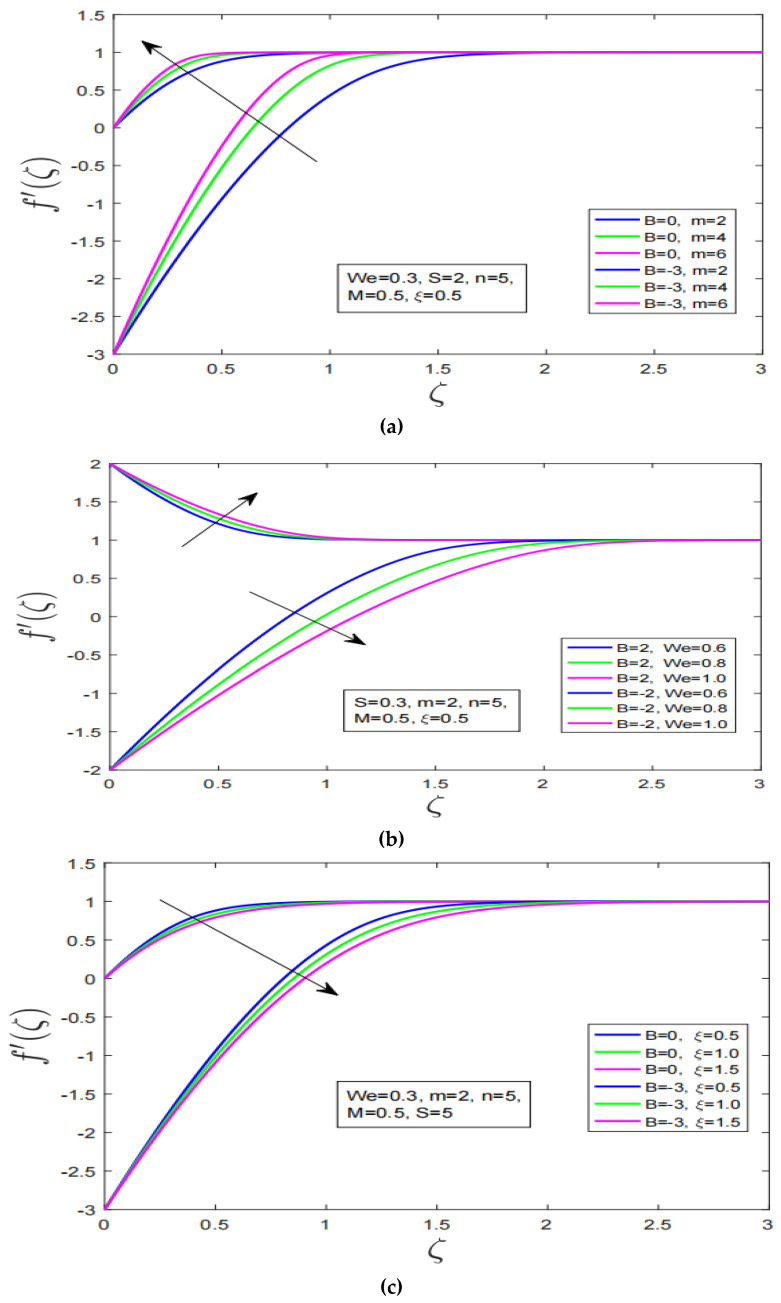

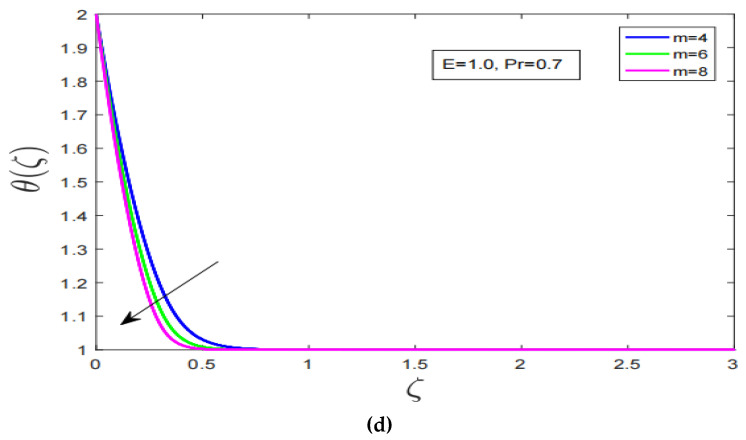
(**a**): Impression of non-linearity effect on velocity profile over stretching and shrinking surface, (**b**): Weissenberg number impact on velocity profile over stretching and shrinking surface, (**c**): Behavior of velocity profile over stretching and shrinking surface under variable viscosity impact, (**d**): Impact of non-linearity effect on temperature profile.

Figure 2 a–c, demonstrates the effect of non-linearity, Weissenberg number and variable viscosity on velocity profile of Carreau fluid for stretching/shrinking surface. Figure 2a, shows that with increment in non-linearity the velocity of Carreau fluid increase for stretching and shrinking. Consequently, the momentum boundary layer expands. Figure 2b, describes the impact of Weissenberg number on velocity profile of Carreau fluid for stretching/shrinking. It is observed that with augmentation in Weissenberg number the velocity of Carreau fluid has decreased for stretching surface. While the motion of Carreau fluid has increased with increment in Weissenberg number for shrinking surface. It is also worth noting here that with change in Weissenberg number dramatically affect the momentum boundary layer of Carreau fluid. More rapid expansion in boundary layer has been observed for shrinking case. Figure 2c, shows the impact of variable viscosity on motion of Carreau fluid over stretching/shrinking surface. It is observed that motion of Carreau fluid decreases over stretching/shrinking surface with increment in viscosity. It is evident from the motion plot that higher viscosity does lower the motion of classical fluid such as Carreau fluid.

**Figure 3:**
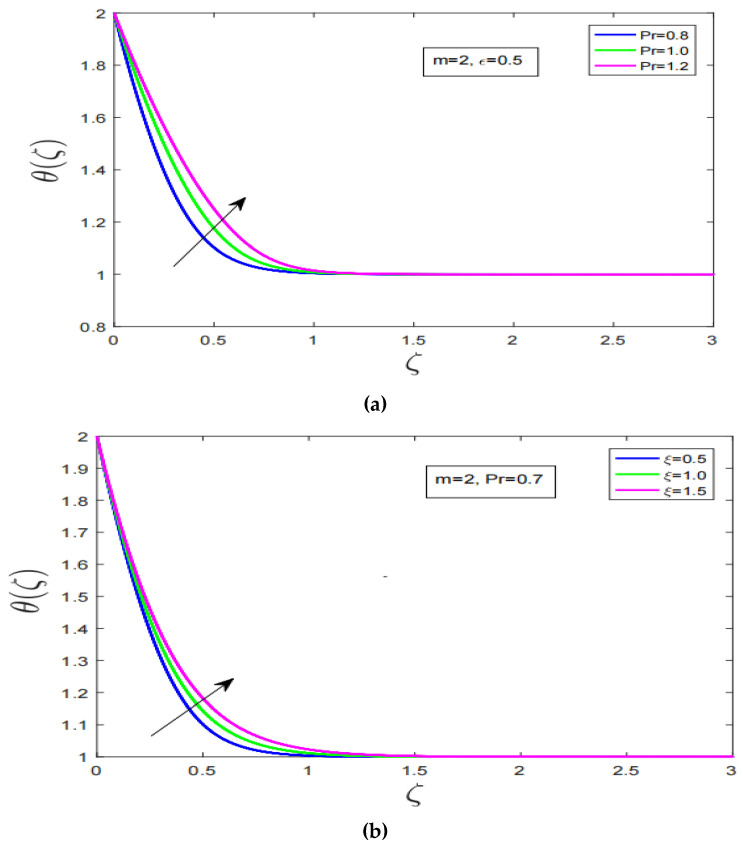

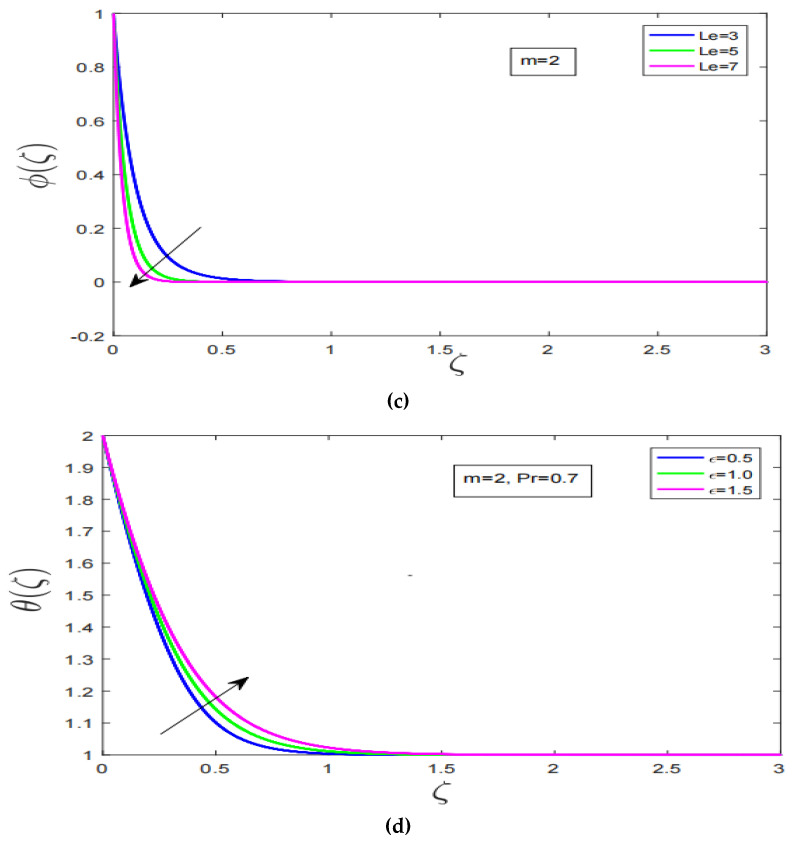
(**a**): Effect of change in Prandtl number on temperature profile, (**b**): Variable viscosity influence on temperature profile, (**c**): Lewis number impact on concentration profile, (**d**): Effect of variable thermal conductivity on temperature profile.

Figure 2d shows the impact of non-linearity effect on temperature profile of Carreau fluid in the event of stretching. Enhancing the non-linearity does sharply decline temperature profile of Carreau fluid. Furthermore, it is worth noting here that when non-linearity is increased associated thermal boundary layer of the Carreau fluid contracts. This phenomenon has occurred due to the presence of strong resistive force in vicinity of boundary layer. Figure 3a,b, elaborate the impact of Prandlt number and variable viscosity on temperature profile, respectively. Figure 3a illustrate the effect of Prandlt number on temperature profile. Prandlt number increase significantly increase the temperature profile. Additionally, higher Prandlt number produces further expansion in thermal boundary layer of the Carreau fluid. Figure 3b, describes the effect of variable viscosity on temperature profile. Although temperature profile has decreased with increment in viscosity of Carreau fluid. The thermal boundary layer of Carreau fluid has increased with increment in viscosity of Carreau fluid.

Figure 3c, discuss the effect of Lewis number on concentration profile. It is observed that with increment in Lewis number the concentration profile decreases sharply. Figure 3d shows the effect of variable thermal conductivity on temperature profile. It is worth noting here that with increment in thermal conductivity temperature profile significantly declines. Furthermore, the thermal boundary layer of the Carreau fluid has expanded with increment in thermal conductivity.

Table 1 provide the results of skin friction and Nusselt number under the varying mass suction effect for stretching/shrinking surface. It observed that in the event of stretching and with increment in mass suction the skin friction and Nusselt number both increases. Whereas, in the event of shrinking with presence of high mass suction force both quantities of physical importance increase. Table 2 elaborates on the varying impact of power law index on stretching/shrinking. It case of stretching with increase in power law index the skin friction has dramatically reduced. Additionally, the Nusselt number under varying impact of power law index has increased. The event of shrinking shows that increase in power law index has effectively enhanced skin friction and decline Nusselt number. Table 3 demonstrate the skin friction and Nusselt number under non-linearity influence for stretching/shrinking. It is concluded that with non-linearity influence high rates of skin friction and the Nusselt number has been obtained.

## 5. Conclusions

In this article, the effect of magneto-hydro-dynamic effect on the Carreau fluid flow over a non-linear stretching/shrinking surface is addressed with convective heat transfer. The variable thermo-physical properties are incorporated. Additionally, flow governing equation are solved numerically with the shooting method in MATLAB and Newton’s method is used to obtain the initial guesses. Moreover, the major outcomes of our study can be summarized as follows:

1. The increment in power law index increases velocity profile for shrinking surface whereas decline has been observed in motion for stretching.
2. High suction reduces the fluid motion for stretching and shrinking. Velocity profile decreased with augmentation in viscosity of fluid. Velocity profile has increased with the increase in Weissenberg number.
3. Increasing the magnetization force has decreased motion of fluid for stretching surface and opposite behavior has been observed for shrinking. Additionally, the increment in highly non-linearity parameter increases the fluid motion for stretching/shrinking.
4. Increment in non-linearity parameter decline the temperature profile sharply.
5. Temperature profile increases with increase in Prandtl number consequently, thermal boundary layer thickness of the fluid expands.
6. Temperature profile has increased with augmentation in viscosity and declination has been observed with increment in Lewis number.
7. Temperature profile is the increasing function of variable thermal conductivity parameter.
8. Nusselt number have increased with increment in non-linearity and suction influence. Minimal shear stress rate is observed under the increasing power law index.

## Figures and Tables

**Table 1:** The numeric results of the shear stress and Nusselt number are under the influence of Stretching ratio and Suction.

Numerical Outcomes of with Fixed Parameters			
M=0.5,n=0.5,m=0.3, We=0.3, ξ=0.5, ϵ=0.5, Pr=0.7			
B	S	$Cf_{x}$	$Nu_{x}$
0	5	2.902	4.8292
0	5.5	3.0122	5.3449
0	6	3.1178	5.8622
0	6.5	3.2191	6.3808
0	7	3.3163	6.9004
0	7.5	3.4098	7.4208
−3	5	4.6210	4.2457
−3	5.5	4.8563	4.8175
−3	6	5.0709	5.3808
−3	6.5	5.2688	5.9377
−3	7	5.4531	6.4897
−3	7.5	5.6258	7.0380

**Table 2:** The numeric results of the shear stress and Nusselt number under the influence of stretching ratio and power law index.

Numerical Outcomes of with Fixed Parameters			
M=0.5,S=5,m=2, We=0.3, ξ=0.5, ϵ=0.5, Pr=0.7			
*B*	*n*	$Cf_{x}$	$Nu_{x}$
2	5	−3.0451	5.0573
2	6	−2.8827	5.0595
2	7	−2.7542	5.0613
2	8	−2.7542	5.0629
2	9	−2.5594	5.0643
−2	5	4.4331	4.4792
−2	6	4.1437	4.4692
−2	7	3.9193	4.4610
−2	8	3.7378	4.4542
−2	9	3.5863	4.4483

**Table 3:** The numeric results of the shear stress and Nusselt number under the influence of stretching ratio and non-linear parameter.

Numerical Outcomes of with Fixed Parameters			
M=0.5,S=5,n=5, We=0.3, ξ=0.5, ϵ=0.5, Pr=0.7			
*B*	*m*	$Cf_{x}$	$Nu_{x}$
0	7	4.4003	13.5537
0	7.5	4.5076	14.4272
0	8	4.6105	15.3008
0	8.5	4.7105	16.3008
0	9	4.8047	17.0482
0	10	4.9856	18.7959
−3	7	6.8340	12.9560
−3	7.5	6.9964	13.8293
−3	8	7.1523	14.7027
−3	9	7.4474	16.4500
−3	10	7.7229	18.1976

## Data Availability

Data can be made available following a request.

## Nomenclature

u,v	Velocities components in respective direction $\left({\mathrm{ms}}^{-1}\right)$	${\left(\rho c_{p}\right)}_{CF}$	Volumetric heat capacity of Carreau fluid $\left({\mathrm{JK}}^{-1}\right)$
$v_{f}$	Kinematic viscosity of Carreau fluid $\left(m^{2}s^{-1}\right)$	$T_{W},\;T_{\infty }$	Surface and Ambient temperature (K)
T,C	Temperature and concentration dimensional profile [−]	$C_{W},\;C_{\infty }$	Surface and Ambient Concentration (K)
$\rho_{f}$	Density of Carreau fluid $\left({\mathrm{kgm}}^{-3}\right)$	ζ	Similarity Variable [−]
$C_{p}$	Heat capacity at constant pressure of Carreau fluid $\left({\mathrm{Jkg}}^{-1}K^{-1}\right)$	$\mu_{CF}$	Dynamic viscosity $\left({\mathrm{kgm}}^{-1}s^{-1}\right)$
*f*	Velocity Profile [−]	D	Mass Diffusivity [−]
θ,ϕ	Temperature and concentration dimensionless profile [−]	$v_{W},\;q_{W}$	Suction/Injection velocity and surface heat flux, respectively [−]
*L* e	Dimensionless Lewis number [−]	$\mu^{*},k_{f}^{*}$	Effect of viscosity and thermal conductivity [−]
$h_{1},\;h_{2}$	Some positive constant	*P* r	Prandtl Number [−]
$N_{1},\;N_{2}$	Some positive constant	ξ,ϵ	Variable viscosity and thermal conductivity parameter, respectively [−]
S	Dimensionless Suction/Injection parameter [−]	$Re_{L}$	Reynolds number [−]
We	Dimensionless Weissenberg number	M	Dimensionless Magnetic field parameter [−]
BLA	Boundary Layer Approximation [−]	B	Stretching ratio parameter [−]
MHD	Magneto-Hydrodynamic [−]		
